# Recent Advances in ADAM17 Research: A Promising Target for Cancer and Inflammation

**DOI:** 10.1155/2017/9673537

**Published:** 2017-11-02

**Authors:** Marcia L. Moss, Dmitry Minond

**Affiliations:** ^1^Verra Therapeutics, 127 Asbury Rd., Lansing, NY 14882, USA; ^2^Rumbaugh-Goodwin Institute for Cancer Research, Nova Southeastern University, 3321 College Avenue, CCR 6th Floor, Fort Lauderdale, FL 33328, USA; ^3^College of Allopathic Medicine, Nova Southeastern University, Fort Lauderdale, FL 33328, USA

## Abstract

Since its discovery, ADAM17, also known as TNF*α* converting enzyme or TACE, is now known to process over 80 different substrates. Many of these substrates are mediators of cancer and inflammation. The field of ADAM metalloproteinases is at a crossroad with many of the new potential therapeutic agents for ADAM17 advancing into the clinic. Researchers have now developed potential drugs for ADAM17 that are selective and do not have the side effects which were seen in earlier chemical entities that targeted this enzyme. ADAM17 inhibitors have broad therapeutic potential, with properties ranging from tumor immunosurveillance and overcoming drug and radiation resistance in cancer, as treatments for cardiac hypertrophy and inflammatory conditions such as inflammatory bowel disease and rheumatoid arthritis. This review focuses on substrates and inhibitors identified more recently for ADAM17 and their role in cancer and inflammation.

## 1. Introduction

ADAM17, as well as many other ADAM family members, is known to process single-spanning membrane proteins such as cytokines, growth factors, receptors, chemokines, and regulators of neurological processes and diseases [1–7]. Currently, there are over 80 substrates processed by ADAM17 and many of them are implicated in cancer and inflammatory conditions. More recently, substrates for ADAM17 have included molecules that are important for tumor immunosurveillance and studying of shedding events orchestrated by this enzyme has led to proposed novel mechanisms of resistance to popular cancer therapies [8–10]. While ADAM17 has a large substrate profile, its activity is typically only activated in response to stimuli that drive disease states [11, 12], making it an attractive target for therapeutic intervention.

Knowledge of the substrates of ADAM17 helped provide a pathway as to what would be the best use of an ADAM17 inhibitor in the clinic. Researchers have now developed selective inhibitors after learning early on of the failures of small molecules that also targeted the matrix metalloproteinase family [13–15]. While the small chemical entities had side effects such as musculoskeletal and liver toxicities [14], they provided proof of concept experiments indicating that targeting ADAM17 would be beneficial for disease conditions such as sepsis and rheumatoid arthritis (RA) [16, 17]. The earlier studies prompted researchers to prepare more selective inhibitors such as the small molecule, INCB7839 [18], and to develop protein therapeutic agents, such as antibodies and the prodomain of ADAM17 [19] that while not orally available, are very target specific. Currently, several of these novel therapeutic agents have entered into the clinic for both cancer and inflammatory diseases.

**Figure 1:**
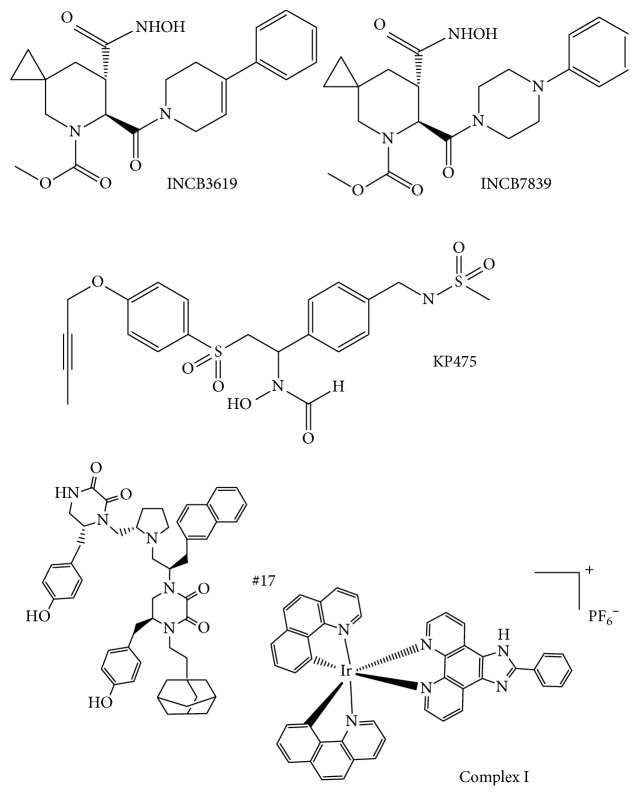
Structures of small molecule inhibitors of ADAM17.

The most advanced is INCB7839, a dual inhibitor of ADAM17 and ADAM10. It is currently being used in the clinic in combination with rituximab for the treatment of diffuse large B-cell non-Hodgkin lymphoma (Figure 1; Table 1) and results should be available in May 2018 (https://clinicaltrials.gov/ct2/show/NCT02141451). Also, recently, an inhibitor of ADAM17 based on its prodomain may be entering into the clinic for inflammatory conditions such as inflammatory bowel disease (IBD) [19]. In this article, we will describe the most recent inhibitors of ADAM17 and also present information on the substrates for ADAM17 that have not been discussed previously in other reviews.

## 2. The Role of ADAM17 in Tumor Immunosurveillance

The function of ADAM17 in tumor immunosurveillance is quite complex and there is a contradictory role for ADAM17 inhibitors to be used for cancer immunotherapy due to the enzyme's substrate profile. In Table 2 are known substrates for ADAM17 that are involved in tumor immunosurveillance.

The first substrate in the table, MHC class I-related chain A and B protein, is a costimulator for cytokine production [20] and was shown to be processed by ADAM10 and ADAM17 using small molecule inhibitors and siRNA treatments [21, 22]. It triggers natural killer cells and certain T cell subsets to have cytotoxic effector activity. The levels of the soluble ligands were detected in sera of patients suffering from multiple types of cancer where they were associated with reduced levels of receptor expression, compromised function of natural killer and cytotoxic cell types, and poor survival [23, 24]. Soluble ligands also inhibited T cell activation and thus contributed to immune escape by tumor cells [21]. Therefore, targeting ADAM17 to reduce soluble ligand levels would increase cellular immune responses to tumor cells.

**Figure 2:**
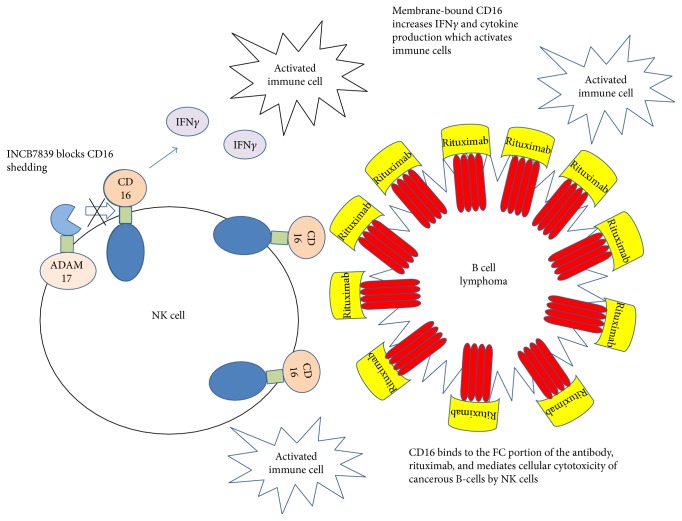
Mechanism of INCB7839 on inhibition of CD16 shedding.

Fc*γ*RIIIA or CD16 is a low-affinity receptor for the IgG expressed on most CD56^dim^ peripheral blood natural killer (NK) cells. Two groups reported that CD16 was a substrate for ADAM17 [25, 26]. Binding of CD16 to antibody-covered cells sent a potent signal to NK cells to eliminate the target and initiate cytokine production such as interferon gamma. Cytokine activation led to a decrease in expression and shedding of CD16. When ADAM17 was inhibited, CD16 shedding was abrogated, and interferon gamma production was increased. ADAM17 inhibition also increased Fc-induced cytokine production of NK cells exposed to rituximab-coated B-cell targets. Rituximab is a monoclonal antibody that targets CD20 on B-cells. It destroys the B-cells and is used to treat diseases such as lymphomas, leukemia, transplant rejection, and autoimmune disorders [27]. In Figure 2 is a mechanism for how targeting ADAM17 can possibly affect the potency of rituximab.

Currently, the ADAM17 inhibitor, INCB7839, is in clinical trials to be used in combination with rituximab for the treatment of diffuse large B-cell non-Hodgkin lymphoma. This is the second time INCB7839 has been used in the clinic for a cancer indication. Previously, it was used to treat patients with HER2-positive breast cancer in combination with trastuzumab [28].

TIM-3, the T cell immunoglobulin and mucin domain-3, is shed by both ADAM17 and ADAM10 [29]. It is highly expressed in exhausted CD8^+^ T cells during chronic infections and individuals with cancer. TIM-3 functions in a similar way to the well-known tumor suppressor, PD-1, or programmed cell death ligand. PD-1 is also expressed on cancer cells currently targeted by the drugs, Keytruda and Opdivo. The drugs work by binding to PD-1 and blocking its function, so that a patient's own immune system is then able to target the cancer cells. This class of drugs is called cancer immunotherapeutics, and it is revolutionizing the way cancer is currently being treated.

Blocking of TIM-3 with monoclonal antibodies reduced PD-1 expression and increased production of cytokines such as IL-12, 6, and 10 [30]. In addition, an antibody specific to TIM-3 also exacerbated symptoms in mice with experimental autoimmune encephalomyelitis, a model for multiple sclerosis. These two findings, taken together, indicate that TIM-3 functions to dampen the immune system [31]. Therefore, an inhibitor of ADAM17 may stimulate the immune system if TIM-3 cleavage is reduced. Mutant mice defective in its shedding will need to be generated in order to understand more fully how cell surface proteolysis relates to the function of TIM-3.

PMEL17 or GP100 is a type I integral membrane protein specifically expressed by melanocytes and melanoma cells that is processed by ADAM17 [32, 33]. PMEL17 is required for the maturation of melanosomes from stage I to II. Release of the soluble form of PMEL17/GP100 can protect tumor cells from antibody-mediated immunity. Investigators have used vaccines targeted against soluble gp100 to treat patients with metastatic melanoma in the clinic. Individuals were immunized with the gp100 peptide vaccine, followed by interleukin 2, and had an objective clinical response of 42% which was higher than just using interleukin 2 alone [34]. The clinical trial results indicate that targeted ablation of soluble gp100 was used successfully to treat melanoma patients. An ADAM17 inhibitor might therefore be useful for melanoma in the clinic, possibly in combination with interleukin 2.

CD154 is a proinflammatory cytokine released from the membrane of T cells by ADAM17 and ADAM10 when it engages CD40 [35]. CD154 is currently being investigated for its role in immunosurveillance in cancer and its function in certain chronic inflammatory and autoimmune diseases. Mouse models have been used to study the function of CD154 in cancer. When an adenovirus carrying the CD154 gene was injected into a variety of established tumors in mice, it produced tumor regression. Also, CD154 gene transfer to T cells injected into mice in several cancer models reduced growth by causing tumor-specific cytolytic T-lymphocyte responses [36]. The significance of membrane-bound versus soluble CD154 is not well understood. However, it is the membrane form that induces an inflammatory response on endothelial cells although the soluble form has been shown to induce B-cell proliferation [37].

CD40 and CD154 interact to modulate T cell priming. Binding to each other also activates NK cells and macrophages and upregulates costimulatory molecules [38]. If an ADAM17 inhibitor blocks CD154 shedding, then, it is likely that the CD154-CD40 interaction would be enhanced, leading to activation of the immune system.

Fc*α*R (CD89) is a receptor for IgA and plays important roles in immune responses, and it is expressed constitutively on monocytes, macrophages, eosinophils, neutrophils, and some types of dendritic cells. It is a substrate for both ADAM17 and ADAM10 [39]. When microorganisms breach the epithelial barrier, they can be cleared through phagocytosis by neutrophils that express CD89 through binding to dimeric IgA [40]. Currently, there are bispecific antibodies (BsAbs) that use the CD89 to target cells with cancer therapeutic agents. Although they have not reached the clinic, these directed BsAbs, using either a HER2 or CD20 in combination with CD89, have proven to be beneficial in preclinical models of cancer [41]. The BsAbs studies underscore the importance of CD89 in tumor immunosurveillance. Since inhibition of ADAM17 has already been shown to improve the efficacy of rituximab [25], the mechanism by which this happens could be due not only to its effect on CD16 but also to its effect on CD89.

The IL23 receptor is a receptor for IL23 and pairs with IL12Rbeta to signal through Janus Kinase 2. ADAM17 depletion was shown to abrogate shedding of IL-23R after PMA stimulation in HEK293 cells [42]. Genetic variants of the IL-23R gene are protective for Crohn's disease and ulcerative colitis, suggesting that the IL-23 receptor function is to suppress the immune system [43]. Growth of tumors is prevented in IL-23 or IL-23R knockout mice, and genetic deletion or antibody-mediated elimination of IL23 increases the activity of cytotoxic T cells in a model of chemically induced carcinogenesis [44]. Again, it is unclear how targeting IL23R shedding via inhibition of ADAM17 would promote tumor inflammatory processes. More likely is that inhibition of IL-23R shedding would lead to immune suppression.

4-1BB belongs to the TNF super family and is expressed on activated T-lymphocytes. It can activate many immune effector cells including CD4 T cells, B-cells, NK cells, monocytes, and macrophages. Agonists, such as a soluble version of 4-1BB called SA-4-1BBL, have antitumor and antiviral activities, indicating that soluble 4-1BB would also have immune-stimulatory properties [45]. Knockdown of ADAM17 in HEK293 cells transfected with recombinant 4-1BB established this enzyme as a sheddase for the TNF super family protein [46]. Increasing cell surface 4-1BB and decreasing soluble levels, with an ADAM17 inhibitor, could lead to suppression of the immune system rather than its activation.

The benefit from the use of an ADAM17 inhibitor to aid other therapeutic agents in cancer immunosurveillance is unclear at this time. However, Incyte is currently using their dual ADAM17 and ADAM10 inhibitors in the clinic to enhance the properties of rituximab, likely because it acts as a stimulator of the patient's immune response to cancer. The use of an ADAM17 inhibitor as a stimulator of one's own immune response seems contradictory. ADAM17 inhibitors can inhibit tumor necrosis factor alpha (TNF*α*) shedding, and early inhibitors had efficacy in rheumatoid arthritis models [14]. Also, there is evidence that targeting ADAM17 for inflammatory diseases such as inflammatory bowel disease (IBD) would be successful. How then could an ADAM17 inhibitor, which has anti-inflammatory properties, be used for cancer immunotherapy?

During early studies with ADAM17 inhibitors, researchers discovered that chemical entities with charged residues could inhibit ADAM17 *in vitro*, but when used *in vivo*, they did not inhibit TNF*α* release in LPS-challenged models [47]. The Incyte inhibitor INCB7839 also does not inhibit TNF*α* release *in vivo*, even though it prevents shedding of many epidermal growth factor receptor (EGFR) ligands that are substrates for ADAM17 [18]. Likewise, the first monoclonal antibody that targeted ADAM17, D1(A12), also does not affect TNF*α* levels *in vivo* while it eliminates TNF*α* release *in vitro* [48]. Thus, these inhibitors may be designed fortuitously, to be used to enhance the properties of other therapeutic agents, possibly by stimulating one's own immune system to fight the cancer.

## 3. ADAM17 Substrates That Mediate Inflammatory Processes

Besides TNF*α* and substrates involved in tumor immunosurveillance, there are over 20 substrates for ADAM17 that are regulators of inflammation. However, only recently reported substrates for ADAM17 will be discussed here as there are many earlier reviews on the subject. In Table 3 is a list of the substrates and their roles in mediating inflammatory processes.

The IL-1 receptor 2 (IL-1R2) is expressed principally on lymphocytes and plays a role in tolerance and immunity of the immune system. IL-1R2 has no cytoplasmic domain and thereby acts as a decoy receptor for IL-1. Soluble IL-1R2, produced by ADAM17 processing, can therefore bind to IL-1 and possibly dampen the immune response [49]. However, treatment with anti-inflammatory agents such as glucocorticoids increased membrane IL1-R2, suggesting that an ADAM17 inhibitor would be anti-inflammatory due to increasing receptor levels of IL1-R2 on the cell surface. Therefore, perhaps, both soluble and membrane-bound forms of IL1-R2 suppress immune-regulatory molecules.

Neogenin belongs to the immunoglobulin family and is a receptor for netrin that is involved in cell survival and axon guidance. It is expressed abundantly in the nervous system. Binding of neogenin to proteins like netrin and other repulsive guidance molecules occurs in the central nervous system and causes inhibition of neurite outgrowth and growth cone collapse while blockage of ADAM17 enhances this inhibition [50]. In addition to the role that neogenin plays in the nervous system, it is also a regulator of inflammatory processes. Acute inflammation is dampened when neogenin is endogenously repressed. Studies using functional inhibition of neogenin resulted in a significant attenuation of inflammatory peritonitis [51]. Neogenin also promotes pulmonary inflammation during lung injury, and its inhibition reduces hepatic ischemia-reperfusion injury [52].

Syndecan-4 (SDC4) is a proteoglycan found on the cell surface of epithelial and fibroblast cells. SDC4 binds to fibroblast growth factors (FGF) and brings them to the FGF receptors on the cell surface. SDC4 is also a mediator of inflammatory responses. Mice deficient in SDC4 exposed to LPS had an increase in neutrophil numbers and CXC chemokines in bronchoalveolar lavage fluid compared to wild-type controls [53]. In addition, administration of SDC4 in the lung caused attenuation of both airway and alveolar inflammation [54]. In contrast, blocking of SDC4 signaling with an antibody reduced eosinophilic airway inflammation in an asthma model using ovalbumin-sensitized mice [55]. It is unclear what effect downregulation of SDC4 processing via ADAM17 inhibition may have on inflammation [56]. However, by increasing SDC4 levels on the cell surface, signaling might be enhanced leading to an exacerbation of asthma.

Glycoprotein VI (GPVI) is a receptor for collagen on platelets and is a protein that is critical for platelet microparticle formation. Platelet microparticles are the most abundant microparticles in the blood. They are 0.1–1 *μ*m fragments shed from plasma membranes of platelets that are important for hemostasis, thrombosis, cancer, and inflammation. Platelet microparticles are found in arthritis as well as in other joint inflammatory disorders like gout, PsA, and juvenile arthritis [57]. Depletion of microplatelets attenuated arthritis in a murine model, and GPVI inhibitors inhibited arthritis and other inflammatory disorders by blocking the activation of platelets, which cause joint inflammation [58]. An ADAM17 inhibitor may be beneficial in arthritis and other inflammatory processes by regulating the glycoprotein's cleavage, thereby preventing microplatelet formation [59, 60]. Recently, a specific inhibitor of ADAM17 was efficacious in a collagen-induced arthritis model, suggesting that targeting the enzyme for arthritis may be beneficial in the clinic [19].

Lymphotoxin alpha beta is a member of the tumor necrosis factor superfamily (TNF-SF) cleaved by ADAM17 [61]. It is a proinflammatory cytokine associated with pathology in rheumatoid arthritis. LTalpha(1)beta(2) heterotrimers are expressed on the surface of activated lymphocytes and induce chemokines, cytokines, and adhesion molecules from primary synovial fibroblasts isolated from RA patients [61]. Lymphotoxin alpha beta is required for differentiation of type I natural killer T (NKT) cells, a lymphocyte subset with important immunoregulatory properties. Type I NKT cells produce TH1 and TH2 cytokines, growth factors, and inflammatory chemokines [62, 63]. It is likely that some of the anti-inflammatory effects of ADAM17 inhibitors and their beneficial effects in RA models may be due to attenuation of lymphotoxin alpha beta shedding.

Clearly, both broad spectrum and now specific inhibitors of ADAM17 can be used to treat inflammatory conditions [16, 19]. While not described in this review, many other substrates for ADAM17 besides TNF*α* also have anti-inflammatory properties [2]. Clinical trials with a specific inhibitor such as the prodomain of ADAM17 which have may begin will determine the fate of using ADAM17 inhibitors for inflammatory conditions. Since ADAM17 processes so many substrates, side effects may occur which would preclude its use for rheumatoid arthritis and inflammatory bowel disease. Current treatments for rheumatoid arthritis such as EMBREL and HUMIRA are quite specific, as they only target TNF*α* [64]. It will be interesting to see how a selective ADAM17 inhibitor performs in such a crowded market.

## 4. Substrates for ADAM17 Associated with Cancer

Initially ADAM17 was identified as a TNF-alpha converting enzyme [6, 7]. However, knockout mice had unique phenotypes. They had open eyes and wavy hair at birth, which was reminiscent of TGF-alpha knockout mice [65]. This led to the discovery that ADAM17 not only cleaves TNF*α* but also TGF-alpha [66]. Subsequently, other members of the EGFR ligand family such as amphiregulin, heparin-binding epidermal growth factor, and epigen were shown to be substrates for ADAM17 [67–69]. As there are many reviews on substrates and cancer, this one will focus on more recent substrates for ADAM17 which are yet to be discussed. In Table 4 are substrates for ADAM17 involved in cancer.

Jagged 1 (JAG1) is a ligand that interacts with Notch receptors, and its presence in cancer is correlated with a poor prognosis [70]. Metastasis, cell proliferation, inhibition of apoptosis, promoting cell survival, and maintaining cancer stem cell populations are all processes regulated by JAG1 via Notch receptor activation. In addition, JAG1 can indirectly affect cancer by influencing tumor microenvironment components such as tumor vasculature and immune cell infiltration. Knockdown of JAG1 by siRNA in several ovarian cancer cell lines decreased cell viability and reduced taxane resistance in one resistant cell line, SKOV3TRip2, *in vitro*. Using *in vivo* xenograft ovarian cancer models, knockdown of JAG1 also reduced tumor size. In addition, siRNA knockdown of JAG1 in SKOV3TRip2 cells *in vivo* also restored taxane sensitivity [71].

Lu et al. found that pretreatment of colorectal cancer cells isolated from patients with endothelial-conditioned media could promote a cancer stem cell phenotype and increase tumorigenicity and metastasis of the cells when injected subcutaneously into mice [72]. Furthermore, soluble factors from the media could activate the Notch pathway and the authors subsequently found that Jagged 1 was present. Depletion of Jagged 1 or blocking with a Jagged 1 antibody reduced the sphere-forming capability of the colorectal cancer cells [72]. Cleavage of Jagged 1 to release a soluble form was carried out by ADAM17 [72, 73]. These findings suggest that endothelial cells can promote cancer stem cell phenotypes through shedding of Jagged 1 by ADAM17.

Glypican-1 (GPC1) is a proteoglycan that plays a role in the control of cell division and growth regulation. In the human pancreatic cancer cell line, PANC-1 cells, downregulation of GPC1 using antisense RNA resulted in slower growth and decreased anchorage-independent growth *in vitro* as well as attenuated tumor growth, angiogenesis, and metastasis *in vivo* when these cells were transplanted into mice [74]. Moreover, mice that lacked GPC1 exhibited decreased tumor angiogenesis and metastasis following intrapancreatic implantation with either PANC-1 or T3M4 human pancreatic cancer cells and had fewer pulmonary metastases following intravenous injection of murine B16-F10 melanoma cells [74].

GPC1 acts as a coreceptor for heparin-binding growth factor (HB-EGF) and fibroblast growth factor 2 (FGF-2) [75]. In a study by Kleef et al., the GPI anchor of GPC1 was removed by PI-PLC generating a soluble form of the protein. Under these conditions, the cells became unresponsive to HB-EGF and FGF-2, and cell proliferation was reduced. In addition, cells were transfected with an engineered transmembrane domain that does not release GPC1, and those cells proliferated in response to HB-EGF and FGF-2 [75]. Using mass spectrometry, Kawahara et al. found that GPC1 is a substrate for ADAM17 [76]. Since the membrane-bound form of GPC1 appears to regulate adhesion, proliferation, and migration of carcinoma cells, downregulation of its shedding with an ADAM17 inhibitor would not likely be advantageous for treating many types of cancer.

Vasorin is a transforming growth factor beta- (TGF*β*-) binding protein and is found in vascular smooth muscle cells. It attenuates TGF*β* signaling *in vitro*. Researchers' studies suggest that one method of attenuation of TGF*β* signaling is through the generation of soluble vasorin. M2 cells, which are mutant and keep ADAM17 in the proform, show an increased response to TGF*β* [77].

Since TGF*β* can act as both a tumor suppressor and activator, it is unclear how inhibition of vasorin shedding could impact cancer progression in humans [77]. However, several lines of research indicate that if the TGF*β*R3 receptor is expressed in the cancer cells, it sequesters TGF*β*, preventing it from signaling. Since this process inhibits tumorigenesis [78], it is likely that inhibition of TGF*β* signaling rather that activation would be beneficial for cancer patients. In addition, because the TGF*β* receptors are all shed, possibly by ADAM17, the net effect on TGF*β* signaling with an ADAM17 inhibitor is difficult to ascertain.

Neuregulin 1 (Nrg1) is necessary for the normal development of the heart and nervous system, and its dysregulation is implicated in schizophrenia [79]. In cancer, overproduction of neuregulins in mammary tissue leads to the generation of adenocarcinomas and increased metastases in breast cancer cells [80]. Lowering of levels of neuregulin by the use of antisense oligonucleotides reduced tumorigenesis and metastasis of breast cancer cells. Furthermore, transmembrane Nrg1 expressed in MCF7 cells activated HER receptors and caused the cells to be sensitized to trastuzumab, a monoclonal antibody that binds HER2 and is used to treat HER2-positive breast cancer. Soluble Nrg1 is also produced by ADAM10 processing and can cause resistance to trastuzumab by binding to cell surface HER3 and inducing compensatory growth factor signaling [81]. More recently, Fleck et al. have discovered that ADAM17 also processes Nrg1 [82].

The trastuzumab findings are significant because using historical data-measuring response rates of the drug, researchers found that many patients who had metastatic breast cancer and who expressed membrane-bound Nrg1 responded well in terms of overall survival and time to disease progression even though they did not express high levels of HER2 [83]. In addition, Nrg1 expression may influence the response to other anti-HER2 strategies, such as treatments based on the monoclonal antibody pertuzumab or the dual small tyrosine kinase inhibitor lapatinib. It is interesting to postulate that an ADAM17 inhibitor may be beneficial for breast cancer patients who are not responding to trastuzumab who also express low levels of HER2 because it would increase membrane-bound Nrg1 and prevent it from binding and activating HER3.

INCB7839, a dual ADAM17 and 10 inhibitor, was used clinically to treat HER2-positive metastatic breast cancer patients in combination with trastuzumab [28]. However, only a subset of the patients seemed to respond which were those individuals expressing the p95 fragment of HER2. Hopefully, future clinical trials will be able to dissect what type of cancer patients will benefit from using emerging ADAM17 therapeutic agents.

Trop2 is a transmembrane glycoprotein overexpressed by various human carcinomas [84, 85]. Its overexpression is correlated with decreased patient survival as well as increased metastases and aggressiveness in many cancers. A Trop2 Fab was identified in a phage display screen that inhibited apoptosis, wound healing, and breast cancer cell growth and proliferation *in vitro*. The same antibody was used *in vivo* in a MDA-MB-231 xenograft model where it inhibited tumor growth and induced apoptosis [86]. Trop2 cleavage was blocked by the ADAM17/10 and MMP inhibitor GW64 as well as by the PKC inhibitor Bim-1 [87]. Therefore, it seems likely that most of the processing was due to ADAM17 because it required PKC activation.

Carbonic anhydrase IX (CA IX) maintains intracellular pH and is necessary for cancer cells to adapt to toxic conditions in the extracellular environment. It is the most widely expressed gene in response to hypoxia. Its expression is correlated with a poor prognosis in several types of cancer and it represents a general marker of tumor hypoxia. Furthermore, the activity of CA IX stimulates the migratory pathways of cancer cells and is connected with the increase of the aggressive/invasive phenotype of tumors [88]. When M2 CHO cells were transfected with CA IX, processing was defective, due to a mutation that kept ADAM17 in its proform. The transfected CHO cells with the M2 mutation were more viable than wild-type cells when exposed to PMA [89]. CA IX shedding was increased in cells undergoing apoptosis from cytotoxic agents such as doxorubicin, suggesting that soluble levels could be used as a biomarker for the effectiveness of cancer-therapeutic agents [90].

Vascular endothelial growth factor receptor-1 (VEGFR-1) or FLT1 is a member of the VEGFR family and binds VEGF-A, PlGF, and VEGF-B. FLT1 has tyrosine kinase activity and its inhibition reduced tumor metastasis even after initial seeding [91]. An anti-FLT1 antibody inhibited tumor angiogenesis, arthritis, and atherosclerosis, suggesting that targeting this receptor may be useful for both inflammatory and cancer-related diseases [92]. VEGF-A stimulated ADAM17 shedding of VEGFR-1, suggesting that processing of the receptor is important for angiogenesis [93].

c-MET is a receptor tyrosine kinase that is principally processed by ADAM10 [94]. However, it was shown recently to also be cleaved by ADAM17 [95, 96]. C-MET activates a wide range of different cellular signaling pathways, including those involved in proliferation, motility, migration, and invasion. Furthermore, there is an amplification of the c-*MET* gene, with consequent protein overexpression and constitutive kinase activation in a number of human primary tumors [97]. In inherited forms of human renal papillary carcinomas and other types of cancer, activating mutations in the c-MET kinase domain were discovered [97].

**Figure 3:**
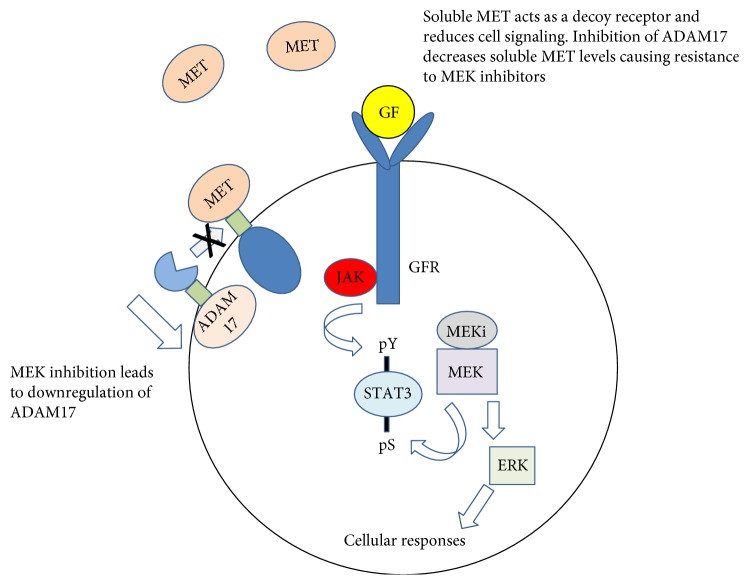
Inhibition of ADAM17 cleavage of MET leads to resistance with MEK inhibitors.

Processing of c-MET is associated with resistance to certain kinase inhibitors [98]. ADAM17 was shown to negatively regulate c-MET signaling by increasing the levels of soluble MET in a KRAS mutant colorectal cancer model [99]. When ADAM17 levels were downregulated by siRNA treatment, the media from cells not only failed to inhibit c-MET activation but actually induced an increase in activation. This result indicates that ADAM17 promotes shedding of soluble MET that inhibits the activation of c-MET. Furthermore, AXL and other ligands and receptors can crosstalk with c-MET and are activated by ADAM17 in the absence of soluble MET. Studies suggest that c-MET activation is a potential mechanism of resistance not only to MEK1/2 inhibitors but also to ADAM17 inhibitors in certain types of cancer (Figure 3). The authors suggest that combination therapies of ADAM17 and c-MET inhibitors would be more clinically effective in KRAS mutant colorectal cancer [99].

The studies with C-MET and other receptor tyrosine kinases indicate that caution must be taken when entering into the clinic and that sometimes, inhibition of shedding can lead to an exacerbation of a disease such as cancer, depending on the tumor microenvironment and the presence or absence of certain signaling molecules. Researchers have found that dual combinations with kinase inhibitors can lead to synergistic effects [98]. Thus, resistance can likely be overcome by administering an ADAM17 inhibitor in combination with these drugs. The proper clinical trial for cancer will likely depend on multiple factors including the cancer type, the cancer's genome, and the drugs that are chosen to be used in addition to an ADAM17 inhibitor.

Protein tyrosine phosphatase alpha (PTP*α*) dephosphorylates Src family tyrosine kinase members and activates them, leading to integrin signaling, cell adhesion, and proliferation. Phosphorylation of Src on tyrosine 789 by PTP*α* promoted integrin-stimulated cell migration. The growth factor IGF-1 also stimulated phosphorylation of PTP*α* to positively regulate cell movement [100]. When ADAM17 is overexpressed in NIH3T3 cells, focus formation was reduced, but there was no change in Src activity [101]. PTP alpha can either induce cellular transformation [102] or suppress it [103]. Therefore, the effect of processing of PTP alpha in cancer is not well understood.

An ADAM17 inhibitor, because of its substrate profile, has so many ways in which to inhibit tumor proliferation. Some of its substrates are growth factors that promote tumorigenesis, while others affect tumor vasculature, cell signaling via inhibition of kinase shedding or regulation of TGF*β* responses. However, ADAM17 also has substrates that act as tumor suppressors such as Klotho and p75 neurotrophin factor [104, 105]. Like most drugs in the clinic, use of an ADAM17 inhibitor will likely have to be patient population specific, depending on which populations will respond. The use of an ADAM17 inhibitor in combination with other drugs will likely prove to be most successful as ADAM17 levels are correlated to resistance to drug therapies [8, 106], possibly by shedding surface molecules for which the drugs bind for efficacy.

## 5. ADAM17 Substrates That Play a Dual Role in Cancer and Inflammation

Some of the same substrates that mediate inflammatory responses are also involved in cancer. Substrates that are important in both of these processes are found in Table 5.

MERTK is a proto-oncogene tyrosine kinase that binds to several ligands including LGALS3, TUB, TULP1, or GAS6. MERTK regulates cell survival, migration, differentiation, efferocytosis, and phagocytosis of apoptotic cells. MERTK also modulates both inflammatory and pro-oncogenic phenotypes. To assess the role of MERTK in tumor models, primary mouse mammary tumor cells derived from female *MMTV-PyVmT* mice were injected into mammary fat pads of MERTK^−/−^ and wild-type mice. The latency period was delayed in the knockout mice relative to controls. Similar findings were observed with C57BL/6-derived B16:F10 mouse melanoma cells injected orthotopically. Lung metastases were also reduced in both of these models [107].

As hematopoietic cells produce MERTK, bone marrow reconstitution systems were also used to assess the effect of MERTK deletion on tumor cells. MMTV-PyVMT mice were either reconstituted with bone marrow from wild-type or MERTK^−/−^ mice, and this revealed reduced tumor volume in the knockout mice. There was an enhanced immune response in the tumor microenviroment of the mice treated with the MERTK^−/−^ bone marrow as well with an increase in the proinflammatory cytokines, IL-6 and IL-12. These findings suggested that the innate immune system was disrupted in the mice bearing the MERTK^−/−^ bone marrow [107].

Macrophages taken from ADAM17 knockout mice were deficient in MERTK shedding, and siRNA treatment to reduce levels of ADAM17 also led to impaired release of MERTK by primary macrophages [108]. Cleavage-resistant mutants of MERTK were made, and the effect was studied in a mouse model of ischemia reperfusion. Mutants have less lung injury, and the inflammation resolution was improved compared to wild-type controls. The resolution in part was helped by long-chain fatty acid-derived lipid mediators which were generated by 5-lipoxygenase resulting from signaling through MERTK [109].

Recombinant soluble MERTK has proinflammatory functions because it suppresses efferocytosis *in vitro* and inhibits thrombus formation *in vivo* [110]. Soluble MERTK is also a decoy receptor for Gas6. Inhibition of Gas6 activity by soluble MERTK caused defective macrophage-mediated engulfment of apoptotic cells. Therefore, an ADAM17 inhibitor that prevented shedding of MERTK with would have anti-inflammatory properties.

The Toll-like receptor-2 (TLR-2) plays a significant role in tumor progression. To assess the role of TLR2 in cancer, a mouse model with a mutation in GP130, *gp130*^F/F^, was used. The *gp130*^F/F^ mutation caused mice to develop gastric tumors. When TLR2 was deleted, the mice had smaller stomachs, smaller gastric lesions, and tumors, compared to just the *gp130*^F/F^ mice. In addition, injection of a TLR2-blocking antibody into *gp130*^F/F^ mice reduced stomach size and tumor burden as well [111]. These results suggest that targeting TLR2 signaling may be beneficial for certain cancer types.

TLR2 is also an activator of innate immunity as it regulates the production of cytokines that are necessary to recognize pathogen-associated molecular patterns (PAMPs) that are expressed on infectious agents. The role of TLR2 was investigated in a model of progressive renal injury. In receptor knockout animals, TLR2^−/−^, there was a decrease in inflammation and influx of neutrophils as well as production of chemokines and TGF*β* in kidneys compared with TLR2^+/+^ animals [112]. TLR2 is also believed to play a role in wound healing in diabetes. In a model of diabetes, TLR2^−/−^ mice showed a decrease in NF-*κ*B and cytokine release and an increase in wound closure [113]. In inflammation, TLR2 is required for recognition of pathogens and for releasing proinflammatory cytokines.

Both ADAM17 and ADAM10 can process TLR2 [114]. Release of TLR2 from either ADAM17- or ADAM10-deficient MEFs was reduced. In addition, the general metalloproteinase inhibitor TAPI-1 or the more specific ADAM10 inhibitor GI254023 both reduced shedding in THP-1 cells. Inhibition of cleavage of TLR2 by ADAM17 may or may not be helpful for either cancer or inflammatory processes as soluble TLR2 ectodomain can negatively regulate TLR2 activation by behaving as a decoy receptor [114].

Syndecan-1 (SDC1) is a proteoglycan found on the surface of epithelial cells and fibroblasts. It binds to fibroblast growth factors (FGFs) and bring them to the fibroblast growth factor receptor (FGFR) on the same cell. Cell surface SDC1 is thought to promote cell adhesion to the ECM and reduce cancer cell migration. For example, head and neck squamous cell carcinoma (HNSCC) cells expressing high levels of SDC1 migrated poorly and are less invasive in collagen I matrices as compared to HNSCC cells expressing lower levels of SDC1 [115]. Knockdown of SDC1 with siRNA in MDA-MB-231 cells reduced cancer stem cell pools significantly and lowered IL6 and IL6R levels which resulted in a decrease in STAT3 signaling [116]. Shed SDC1 was correlated with a poor prognosis and resistance to many chemotherapeutic agents [117]. SDC1 also enhanced oncogene and growth factor signaling, inhibited cancer cell apoptosis, and promoted angiogenesis. Both specific inhibitors and shRNA were used to show that processing of SDC1 occurred by ADAM17 [56].

SDC1, which is an important protein in regulating tumor migration, also is used to generate a chemotactic gradient for immune cells. In a mouse model of bleomycin-induced acute lung injury, SDC1 shedding generated a CXC chemokine gradient that directed the transepithelial migration of neutrophils and attenuates lung inflammation induced by intranasal administration of allergens [118].

Insulin-like growth factor receptor 1 (IGFR-1) is a mediator of both cancer and inflammation. It is a receptor for IGF, and IGF is considered a promoter of tumor progression. Inhibition of IGFR with the inhibitor NT157 caused a substantial reduction in tumor burden by affecting cancer cells, cancer-associated fibroblasts (CAF), and myeloid cells. Decreased cancer cell proliferation and increased apoptosis were accompanied by inhibition of CAF activation and decreased inflammation [119].

IGF-1, through binding to the IGFR-1 receptor, stimulates T regulatory cells and is efficacious in experimental models of multiple sclerosis. T regulatory cells maintain tolerance to self-antigens and prevent autoimmune disease. In IGFR-1^−/−^ mice, the benefits of IGF-1 administration are not observed [120]. These findings suggest that IGFR-1 is a mediator of inflammatory processes.

ADAM17 is involved in the processing of IGFR-1 as knockdown of the enzyme leads to decreased shedding of this growth factor receptor [121]. IGFR-1 cleavage resulted in generating a fragment which ends up in the nuclei of cells. The fragment was obtained due to ADAM17 processing and further cleavage by intramembrane proteinases [121].

The leucine-rich repeats and immunoglobulin-like protein 1 (LRIG1), which is a negative regulator of EGFR, are tumor suppressors that inhibit receptor tyrosine kinases and may be related to chemoresistance. In U251 multidrug-resistant glioma cells, an increase in expression of LRIG1 caused them to be more sensitive to temozolomide, a chemotherapeutic agent [122]. In addition, adenovirus-mediated LRIG1 expression enhanced the chemosensitivity of bladder cancer cells to cisplatin [123]. The broad spectrum metalloproteinase inhibitor TAPI-1 inhibited LRIG1 processing, and overexpression of ADAM17 stimulated its cleavage [124]. Administration of the shed portion of LRIG1 to cells inhibited EGF signaling suggesting that processing is necessary for this substrate to act as a tumor suppressor [124].

LRIG1 is also a regulator of the STAT-3-dependent inflammatory pathway. LRIG1^−/−^ mice suffer from corneal blindness probably through activation of STAT3. Inhibition of STAT3 rescued the LRIGI^−/−^ phenotype [125]. Therefore, as in protumorigenic models, LRIG1 acted as a suppressor of inflammatory processes as well.

## 6. Novel Therapeutic Agents That Target ADAM17

As mentioned in previous sections, ADAM17 is involved in shedding of multiple cell surface molecules which means that it is involved in regulation of multiple cellular processes, both pathological and normal. Therefore, one of the biggest challenges in developing agents inhibiting ADAM17 is to attain selective inhibition of pathological processes while sparing normal processes in order to avoid adverse effects. Another challenge is to develop selective agents against its closest isoform, ADAM10, and other metzincins.

In this section, we will review main molecular types of agents targeting ADAM17 that have been developed in recent years.

## 7. Small Molecules

Incyte Corp. has developed multiple inhibitors of ADAM17, some of which were tested in clinical trials (Figure 1). In early studies, INCB3619, a potent inhibitor of ADAM17 and ADAM10, was tested in several preclinical xenograft models [126]. In a nonsmall lung cancer model, INCB3619 decreased tumor growth and enhanced the therapeutic benefit of paclitaxel. It also blocked the release of heregulin and made the cells sensitive to the EGFR inhibitor, gefitinib. In addition, INCB3619 worked alone and synergized with paclitaxel to block growth of breast cancer in a xenograft model [126].

As stated earlier, INCB7839 (aderbasib), a dual low-nanomolar hydroxamate-based inhibitor of ADAM10 and 17, which was discovered via extensive medicinal chemistry program [127–131] targeting shedding of HER2 in HER2^+^ breast cancer, was discovered. Shedding of HER2 by ADAM10 and 17 may lead to drug resistance for HER2-targeting drugs trastuzumab [8] and lapatinib [18]. INCB7839 was tested at 30 mg/kg/d in BT474-SC1 *in vivo* breast cancer xenograft model in combination with 75 mg/kg of lapatinib which led to the complete prevention of increase of mean tumor volume [18]. Subsequently, INCB7839 was tested in a breast cancer clinical trial in combination with trastuzumab (Herceptin), but was discontinued despite initial promise [28]. The discontinuation was likely because it caused an increase in deep vein thrombosis in a number of patients. INCB7839 in combination with trastuzumab increased the response rate in HER2-positive metastatic breast cancer patients with advanced disease, relative to historic controls (50% versus 15–35%). The response rate was even higher, 64% when the plasma concentrations of INCB7839 were above the IC50 for HER2 cleavage [132].

INC7839 also improved progression-free survival in a subset of the patients expressing the p95 fragment of HER2. The p95 fragment is a constitutively active portion of HER2 left behind on cells once HER2 is cleaved and its presence leads to trastuzumab resistance. It is also an indicator of poor prognosis and survival [133]. INCB7839 in combination with trastuzumab also improved the response rate and overcame trastuzumab resistance in the p95-positive HER2-positive subpatient population. The combination was generally safe and well tolerated [134]. The inhibitor is currently in clinical trials for use in combination with rituximab for large diffuse non-Hodgkin B-cell lymphoma. Trial results are expected in May 2018.

A novel reverse hydroxamate-based selective small molecule inhibitor of ADAM17, KP457 [135], was recently reported (Figure 1). It has low nanomolar activity against ADAM17 in biochemical assay and is more than 70-fold less potent against ADAM10 and MMPs (Table 2). It prevented shedding of glycoprotein Ib*α* (gIb*α*, the Von Willebrand factor receptor critical for adhesive function and platelet lifetime *in vivo*) in human-induced pluripotent stem cell-derived platelets. This resulted in enhancement of the production of functional human iPSC-derived platelets at 37°C, suggesting that ADAM17 inhibition can be important in production of other stem cell-derived cell types with potential clinical applicability.

A series of ADAM17-selective exosite-binding inhibitors was developed as a result of utilization of exosite-binding glycosylated substrate [136]. Several of the lead compounds have been characterized for biochemical and biological *in vitro* potency and selectivity against synthetic [137] and native substrates [138] of ADAM17. The two most potent inhibitors, *17* and *19*, [138] exhibited low micromolar potency and high selectivity for ADAM17 (Table 1). Additionally, compound *17* exhibited unusual substrate selectivity by sparing ADAM17-mediated cleavage of TGF*α*. For comparison, both marimastat (a broad spectrum ADAM and MMP zinc-binding inhibitor) and INCB84298 (a moderately ADAM17-selective zinc-binding inhibitor) inhibited shedding of each tested EGFR ligand (heregulin, TGF*α*, HB-EGF, AREG, and EGF) in A549 cells almost in an equipotent manner [126]. Even though limited enzyme selectivity might be achieved by targeting the zinc of the ADAM17-active site, the zinc-binding inhibitors cannot selectively inhibit proteolysis of the subset of ADAM17 substrates. These inhibitors are currently being advanced into preclinical studies.

A novel iridium(III)-based cyclometalated complex was reported to be an inhibitor of ADAM17 with an IC_50_ value of 29 *μ*M [139] in the biochemical synthetic substrate assay (Figure 2). It also exhibited an 11 *μ*M IC_50_ value for inhibition of LPS-induced shedding of TNF*α* as well as low micromolar inhibition of phosphorylation of p38 in THP-1 cells. Unfortunately, no data on selectivity of this complex towards other metzincins were reported.

## 8. Antibodies

One of the most innovative developments of recent years in ADAM17 inhibition field is an antibody D1(A12) binding to both catalytic and noncatalytic domains of ADAM17 [140]. D1(A12) exhibited subnanomolar IC_50_ in biochemical assay using synthetic substrate and was not active up to 1000 nM against ADAM10 [140]. Additionally, it inhibited shedding of cognate substrates of ADAM17 (TNF*α*, TGF*α*, AREG, HB-EGF, and tumor necrosis factor receptor 1 (TNFR1) with low nanomolar potency in cell-based assays.

D1(A12) demonstrated suitable pharmacokinetics at 10 mg/kg i.p. dosing (nontumor-bearing mice: *C*_max_ = 523 ± 58 nM, *T*_max_ = 2 days, and *t*_1/2_ = 8.6 days) and IGROV1-Luc tumor-bearing mice (*C*_max_ = 425 ± 51 nM) [48]. To measure *in vivo* efficacy, D1(A12) was dosed at 10 mg/kg i.p. in mice bearing IGROV1-Luc tumor (*n* = 11), which reduced average tumor burden by 44% as compared to vehicle control. Analysis of concentrations of ADAM17 substrates in plasma and ascites revealed a 4.4-fold decrease of soluble TNFR1*α*, 5.4-fold decrease of soluble AREG and 15-fold decrease of soluble TGF*α* in ascites suggesting that smaller size of tumors in mice treated with D1(A12) was possibly due to inhibition of EGFR signaling and increase of TNFR signaling.

D1(A12) was also tested against triple-negative breast cancer (TNBC) *in vitro* and *in vivo* [141]. In the clonogenic 2D cell viability assays with HCC1937 and HCC1143 cells, the results were cell line dependent. With HCC1937, the monoclonal antibody reduced both the number of colonies and the total colony area. However, with HCC1143 cells, D1(A12) attenuated the total colony area, but had no effect on colony number. In 3D cell culture, D1(A12) was also found to reduce cell growth of both HCC1143 and HCC1937 cells. In both 2D and 3D cultures, the cell viability was diminished due to the induction of apoptosis as a result of inhibition of TGF-alpha shedding which decreases EGFR signaling. Treatment of HCC1937 cells with D1(A12) significantly reduced cell invasion through an extracellular matrix by 43.2% and migration of HCC1143 by 48.5%.

Similarly, in head and neck squamous cell carcinoma (HNSCC) cells *in vitro*, D1(A12) suppressed proliferation and motility in the absence or presence of the EGFR tyrosine kinase inhibitor (TKI) gefitinib. D1(A12) decreased both the endogenous and the bradykinin- (BK-) stimulated shedding of EGFR ligands, accompanied by a reduction in the phosphorylation of HER receptors and downstream signaling pathways including STAT3, AKT, and ERK [142].

D1(A12) does not cross-react with murine ADAM17, thereby limiting its usefulness and development as a cancer therapeutic agent. Therefore, further experimentation was done to develop an antibody that both cross-reacted between human and murine species, but also retained potency against ADAM17. Towards this end, a novel antibody, A9(B8), was developed through phage display selection studies. A9(B8) was tested *in vitro* for inhibition of amphiregulin (AREG) shedding in two tumor cell lines, PC3 (human prostate), and DT8082 (mouse pancreatic cancer). After PMA or ionomycin stimulation, AREG was released and the effect on shedding with A9(B8) was determined. The IC_50_ value for inhibition of shedding after PMA activation was 200 and 250 nM in PC3 and DT8082, respectively. In DT8082 cells, gemcitabine upregulates ADAM17 at the protein level and enhances AREG shedding. A9(B8) significantly inhibited AREG shedding after gemcitabine treatment, indicating as well that this antibody warrants further preclinical evaluation using *in vivo* studies [143]. More recently, A9(B8) delayed tumorigenesis in a PdxCre;Kras(G12D;Trp53(fl/+) pancreatic ductal adenoma cancer model [144]. Therefore, ADAM17 inhibition may be beneficial for pancreatic cancer.

The A9(B8) antibody was tested in a mouse model of cardiac hypertrophy where they were infused with angiotensin 2 for two weeks [145]. The antibody prevented endoplasmic reticulum stress and cardiovascular remodeling, but had no effect on hypertension induced by the angiotensin 2. Thus, ADAM17 inhibitors may be useful for the treatment of certain hypertensive conditions.

**Figure 4:**
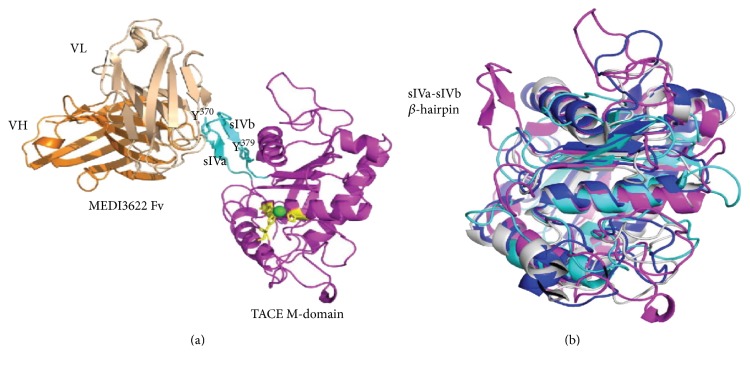
MEDI3622 binds to the unique loop in the ADAM17 structure. (a) Modeling of binding of MEDI3622 to ADAM17. sIVa-sIVb loop is shown in cyan. (b) Overlay of ADAM17 (magenta), ADAMTS-5 (blue), MMP-9 (cyan), and ADAM22 (gray) structures shows that the sIVa-sIVb loop does not align with structures of other metzincins suggesting its uniqueness. Adopted with permission from [154].

Another ADAM17 inhibitory antibody (MEDI3622) was recently developed and studied *in vitro*, ex vivo, and *in vivo* [146, 147]. Binding and molecular modeling studies suggest that MEDI3622 interacts with a unique hairpin loop in ADAM17 structure which is absent in ADAMs, ADAMTSs, and MMPs forming a basis of its isoform specificity (Figure 4, permission available). This binding mode is different from D1(A12), which binds both catalytic and noncatalytic domains [140] and TIMP-3, which binds the catalytic domain. *In vitro* biochemical potency was comparable to D1(A12) (IC_50_ = 3.1 and 4.5 nM, resp.) and it inhibited shedding of various ADAM17 ligands in cell-based assays with low nanomolar potency. Overall, 15 xenograft models were evaluated for tumor growth inhibition (ΔTGI) for MEDI3622 efficacy alone and in combination with cetuximab.

Tumor lysates were additionally evaluated for EGFR phosphorylation. MEDI3622 demonstrated a dose-dependent increase in antitumor activity in OE21 esophageal xenograft model, with 30 mg/kg dose yielding a tumor growth inhibition (ΔTGI) of 102%. The combination of MEDI3622 with cetuximab led to complete tumor regression in all mice. MEDI3622 also showed a strong effect in HNSCC xenograft Cal27 (ΔTGI = 112) and colorectal cancer model H292 (ΔTGI = 110). These three most responsive to MEDI3622 models have the greatest levels of p-EGFR at Y^845^, Y^1086^, and Y^1173^. Esophageal OE21 tumors in particular are hyperphosphorylated at all of these sites compared to any other tumor model. These three also appeared to have the highest total EGFR levels.

## 9. Prodomain

There were several instances of utilization of ADAM17 prodomain as ADAM17 inhibitor in the past [148–151]. Recently, a furin cleavage-resistant version of ADAM17 prodomain (R^58^A, R^211^A, R^214^G, and C^184^A, referred to herein as 4mut) was described [19, 152]. 4mut exhibits 119 nM IC_50_ for ADAM17 inhibition and good selectivity for ADAM17 against MT1-MMP, MMP9, and MMP7 (IC_50_ > 1.0 *μ*M) (Table 1).

The inhibitory activity of 4mut was tested in a cell-based assay using two types of cell lines: CHO cells stably transfected with human TNF*α* and primary macrophages harvested from balb/c mice. 4mut exhibited a-dose dependent inhibition of TNF*α* shedding in CHO cells. A concentration of 5 *μ*M reduced secretion of TNF*α* by 5-fold as compared to no treatment. Treatment with 4mut prevented TNF*α* secretion by 6-7-fold at the highest concentration (1 *μ*Μ).

The therapeutic potential of 4mut was also evaluated in inflammatory disease models.

At 4 mg/kg 4mut decreased levels of TNF*α* ~10-fold (from 3 *μ*g/mL to 0.35 *μ*g/mL) in a LPS-induced C57/BL mouse sepsis shock model. In the DBA/lLacJ mouse model of collagen-induced arthritis, mice treated with 4mut displayed a significantly lower arthritis severity index score, histological score, and serum antibodies specific to type II collagen in a concentration-dependent manner.

The patients are currently being recruited for clinical trials of 4mut in Crohn's disease, ulcerative colitis, septic shock, rheumatoid arthritis, systemic lupus erythematosus, and type II diabetes.

## 10. Regulation of ADAM17 Activity

There have been several breakthroughs in deciphering mechanisms for the way in which ADAM17 is regulated. Recently, researchers discovered that genetic deletion of iRHOM2, an inactive rhomboid proteinase, in mice, prevents TNF-alpha shedding [153]. They discovered that iRHOM2 is a binding partner for ADAM17. The inactive rhomboid is necessary for ADAM17 maturation where it is required for transport of ADAM17 from the endoplasmic reticulum (ER) to the plasma membrane [153].

Subsequently, it was determined that iRHOM2 plays a role in the substrate specificity of ADAM17. Mouse embryonic fibroblasts (MEFs) that lack iRHOM2 were tested for their ability to process multiple ADAM17 substrates. While they were unable to process TNF-alpha, and many EGF ligands such as heparin binding growth factor and amphiregulin in response to stimuli, the shedding of TGF-alpha, CD62L, or ICAM-1 was unaffected [154]. In contrast, knockout of both iRHOM2 and another inactive rhomboid, iRHOM1, ablated all ADAM17-dependent cleavage events [155]. The mice with deletions of the iRHOM1 and iRHOM2 genes resembled the ADAM17 knockout mice as the deletions were embryonic lethal and the mice had open eyes and growth plate defects and misshapen heart valves at birth [155]. Since EGF ligand knockout mice also have these defects, these findings indicate that the inactive rhomboid proteinases are required for processing of EGF ligand family members.

Since the discovery of ADAM17, scientists knew that PMA could activate the enzyme. However, it was unclear until recently what role protein phosphorylation has played. Dang and coworkers determined early on that substrate selection by ADAM17 utilizes different kinases such as PKC-*α*, PPP1R14D, and PKC-*δ* [156]. The N-terminus of iRHOM2 is required for substrate processing by ADAM17. Grieve et al. recently determined that its phosphorylation is necessary and is critical for shedding to occur [157]. A triple serine mutant was made in the N-terminus of IRHOM2 that could no longer be phosphorylated. Cells that lacked iRHOM2 were transfected with the triple serine mutant, and maturation of and processing by ADAM17 was investigated. While ADAM17 was transported from the ER to the cell surface normally, the activity of ADAM17 was impaired. The researchers went on to demonstrate that 14-3-3 proteins bind to the phosphorylated iRHOM2 and releases ADAM17 where it becomes active. Therefore, one of the functions of iRHOM2 is to keep ADAM17 in an inactive state [157].

## 11. Future Directions and Conclusion

ADAM17 has been described as the enzyme that does it all. With over 80 known substrates now reported to be processed by ADAM17, clearly, the enzyme plays a major role in the areas of cancer and inflammation. The substrates range from molecules important in tumor immunosurveillance to those affecting microparticle platelet formation. With the discovery of novel and specific therapeutic agents, this enzyme is an important target not only for rheumatoid arthritis, sepsis, and other inflammatory diseases where it was initially implicated but also for many aspects of cancer therapy and possibly even cardiac hypertrophy. Unfortunately, the failures of all the previous small molecule inhibitors that reached the clinic have discouraged drug companies from progressing the new therapeutic agents which specifically target ADAM17 from entering into clinical trials. Also, since ADAM17 processes multiple substrates, the perception is that even specific inhibitors of this enzyme would have many unwanted side effects. However, the Incyte inhibitor, INCB7839, that targets ADAM10 and ADAM17 and thus almost all shedding events, was in phase II clinical trials where deep vein thrombosis was the only reported side effect. In addition, this inhibitor has re-entered into the clinic for large diffuse non-Hodgkin B-cell lymphoma. Inhibition of ADAM17 may have deleterious consequences for long-term administration, as it played a protective role in the intestinal mucosa in a mouse model of inflammatory bowel disease [158, 159]. Nevertheless, targeting ADAM17 with a selective inhibitor still seems a viable and attractive approach for use in the clinic for cancer indications.

Researchers are now also focusing on specific inhibitors that target only a subset of the shedding events performed by ADAM17. Toward this end, as noted above, exosite-binding inhibitors have been made that spare TGF-alpha processing but inhibit other cellular shedding events. In addition, the interactions between iRHOM2 and ADAM17 may possibly be targeted as mice who have inactivation of the iRHOM2 gene have tissue-specific prevention of maturation of ADAM17 in myeloid cells. The other inactive rhomboid, iRHOM1, is found most everywhere but myeloid cell where it regulates the maturation of ADAM17. Mice, lacking the iRHOM2 gene, were protected in an inflammatory arthritis model, suggesting that inhibitors that target the interaction between iRHOM2 and ADAM17 could be used to selectively treat inflammatory diseases [160]. The field awaits clinical trials for which results will hopefully demonstrate that ADAM17 inhibitors can be turned into useful drugs.

## Figures and Tables

**Table 1:** Summary of biochemical selectivity testing of ADAM17 inhibitors against a panel zinc of metalloproteases. Synthetic substrates were used for all assays. All results are IC_50_, *μ*M.

Lead	MMP1	MMP2	MMP8	MMP9	MMP12	MMP14	ADAM33	ADAM9	ADAM10	ADAM17
Number 17 [136]	NT	NT	>100	NT	NT	>100	NT	NT	>100	4.2
Number 19 [136]	NT	>100	>100	>100	NT	>100	NT	NT	>100	4.3
KP-457 [135]	>100	0.72	2.2	5.4	NT	2.14	NT	NT	0.75	0.011
INCB3619 [18]	>0.5	0.045	NT	0.3	NT	0.8	1.03	>5	0.022	0.014
Complex I [139]	NT	NT	NT	NT	NT	NT	NT	NT	NT	11
MEDI3622 [147]	NT	NT	NT	NT	>10	NT	NT	NT	>10	0.0031
D1(A12) [140]	NT	NT	NT	NT	NT	NT	NT	NT	>1	0.0045
D8 [143]	NT	NT	NT	NT	NT	NT	NT	NT	NT	0.0012
4mut [19, 152]	NT	NT	NT	>1.0	NT	>1.0	NT	NT	NT	0.1

NT: not tested.

**Table 2:** Substrates implicated in tumor immunosurveillance.

Substrate	Tumor immunosurveillance
MHC class I-related chain A and B protein [21, 22, 161]	Costimulator of cytokine production. Triggers cytotoxic effector activity of natural killer cells and certain T cell subsets. Immune escape if shed

Fc*γ*RIIIA (CD16) [25, 26]	Prevention of shedding improves NK cell and improves antibody-dependent cellular toxicity (ADCC) of rituximab

Tim-3 [29, 162]	Blocking with monoclonal antibodies and posterior stimulation with TLR results in an increase in the production of IL-12, IL-6, and IL-10, but a reduced expression of PD-1, an inhibitor molecule of the T cell function [30]
	Treatment with antibody specific to TIM-3 exacerbated experimental autoimmune encephalomyelitis (EAE) in mice and increased the proliferation and activation of macrophages [31]

PMEL17 [33]	Patients with metastatic melanoma immunized with the gp100:209–217 (210 M) followed by high-dose interleukin 2 lead to a better response rate than interleukin 2 alone [34]
GP100 [32]	

CD154 [35, 163]	Injection of adenovirus CD154-established tumors produced sustained tumor regression in the majority of tumor-carrying mice. CD154 gene transfer elicited a tumor-specific cytolytic T-lymphocyte response that suppressed the growth of established lung carcinoma in the presence of IFN*γ* [36]

Interleukin 23 receptors [42]	Genetic deletion or antibody-mediated elimination of IL-23 leads to increased infiltration of cytotoxic T cells rendering a protective effect against chemically induced carcinogenesis. Transplanted tumors are growth-restricted in hosts depleted for IL-23 or in IL-23-receptor-deficient mice [44]

4-1BB [46]	Agonists can both potentiate antitumor and antiviral immunity, while at the same time ameliorating autoimmune disease [164]
	Preclinical data with agonist, SA-4-1BBL, mediates stimulation for tumor immunotherapy [45]

**Table 3:** Substrates involved in inflammation.

Substrate	Role in inflammation
IL-1 receptor2 [49]	A decoy receptor that binds IL-1A, IL-1B, and IL-1Ra

Neogenin [50]	Endogenous repression of neogenin demonstrates attenuated changes of acute inflammation and inflammatory peritonitis [51]
	The guidance receptor neogenin promotes pulmonary inflammation during lung injury [52]
	Inhibition of neogenin dampens hepatic ischemia-reperfusion injury [165]

Syndecan-4 [56]	Using SDC4^−/−^ mice and an antibody that inhibits SDC4 signaling, airway inflammation was reduced in a murine asthma model [55]

Glycoprotein VI (GPVI) [59, 60]	Blockade or antibody-mediated depletion in circulating platelets was shown to effectively inhibit experimental thrombosis, and thromboinflammatory disease states, such as stroke, discuss the potential use of anti-GPVI agents to treat these pathologies in humans [166]
	Collagen receptor glycoprotein VI is a key trigger for platelet microparticle generation in arthritis pathophysiology [58]

LTalphabeta (lymphotoxin alpha beta) [61]	Proinflammatory cytokine associated with pathology in rheumatoid arthritis. Lymphotoxin alpha beta is required for differentiation of type 1 natural killer T (NKT) cells, a lymphocyte subset with important immunoregulatory properties [62, 63]

**Table 4:** Substrates with a role in cancer.

Substrate	Role in cancer
Jagged 1 (JAG1) [72, 73]	JAG1 is a ligand for Notch receptors and is a target for colon, breast, cervical, ovarian, and hematological cancers [167].

Glypican-1 (GLP1) [76]	Downregulation of GPC1 in pancreatic cancer cells resulted in attenuated tumor growth, angiogenesis, and metastasis *in vivo*. Angiogenesis and metastasis were decreased in GPC1 knockout mice following intrapancreatic implantation with human pancreatic cancer cells [74].

Vasorin [77]	Vasorin directly binds to transforming growth factor (TGF) *β* and attenuates TGF*β* signaling *in vitro*. Overexpression of vasorin leads to the attenuation of TGF*β* signaling through the generation of the soluble form.

Neuregulin 1 [82]	Neuregulins are expressed in a significant subset of patients with breast cancer and their presence correlates with clinical response to certain antitumoral treatments such as trastuzumab [83].
	Overexpression of neuregulins in the mammary tissue results in the generation of adenocarcinomas in MCF7 breast cancer cells. In addition, reduction of neuregulin by the use of antisense oligonucleotides reduces tumorigenesis and metastasis [80].

Trop2 [87, 168]	Trop2 is upregulated in tumors and correlates with increased aggressiveness and metastasis [84, 85].
	Trop2 Fab inhibits the growth of breast cancer xenografts [86].

Carbonic anhydrase IX(CA IX) [89, 90]	CA IX is the most widely expressed gene in response to hypoxia and is connected with the increase of the aggressive/invasive phenotype of tumors [88].
	Its expression is closely related to prognosis of the clinical outcome in several tumor types [88].

FLT-1 [93] vascular endothelial growth factor receptor 1 (VEGFR1)	FLT1 inhibition reduces tumor metastasis, suggesting that it represents a therapeutic target in metastatic disease [91].
	Inhibition of tumor angiogenesis, arthritis, and atherosclerosis by anti-Flt1 [92].

C MET receptor [95, 96]	Activating mutations were discovered in the c-MET kinase domain in both sporadic and inherited forms of cancer [97].
	CMET is involved in proliferation, motility, migration, and invasion [169, 170].

Protein tyrosine phosphatase alpha (PTP*α*) [101]	PTP*α* promotes integrin-stimulated cell migration. PTP*α* binds to RACK1, which is a molecular scaffold protein that integrates integrin and growth factor signaling. Binding stimulates IGF-1 to phosphorylate PTP*α* to promote cell migration [100].

**Table 5:** Substrates implicated in both cancer and inflammation.

Substrate	Role in cancer	Role in inflammation
MERTK [108]	MERTK inhibition via monoclonal antibodies, ligand traps, or small molecule tyrosine kinase inhibitors can reverse pro-oncogenic phenotypes [171].	Cleavage-resistant mutants of MERTK have less lung injury and inflammation in a reperfusion injury model [109].

Toll-like receptor 2 (TLR2) [114]	In a cancer model, mice lacking *TLR2* had smaller stomachs, smaller gastric tumors, and fewer gastric lesions compared with control mice. Also, injection of a TLR2-blocking antibody into mice reduced stomach size and tumor burden using mice with gastric cancer [111].	TLR2^−/−^ mice compared with TLR2^+/+^ animals, using a model of progressive renal injury, had reduced inflammation and influx of neutrophils and production of chemokines and TGF*β* in kidneys [112].
		In a diabetic wound model, the absence of TLR2 results in decreased inflammation and improved wound healing [113].

Syndecan-1 (SDC1) [56]	Soluble SDC1 is correlated with a poor prognosis and resistance to many chemotherapeutic agents [117].	In a mouse model of bleomycin-induced acute lung injury, SDC1 shedding attenuates lung injury [118].

Insulin growth factor 1 receptor (IGF1R) [121]	Increased IGF1R expression has been reported in several cancers [172, 173]. NT157, which targets IGF1R, reduces tumor burden [119].	IGF-1, through binding to IGF1R, is a suppressor of the immune system and has been used in experimental models of multiple sclerosis [120].

Leucine-rich repeats and immunoglobulin-like domains 1 (LRIG1) [124]	LRIG1, human EGFR inhibitor, reverses multidrug resistance [174].	LRIG1 is a negative regulator of the STAT3-dependent inflammatory pathway [125].
	Adenovirus-mediated LRIG1 expression enhances the chemosensitivity of bladder cancer cells to cisplatin [123].	

## Abbreviations

ADAM: A disintegrin and metalloproteinase
AREG: Amphiregulin
BK: Bradykinin
CA IX: Carbonic anhydrase IX
EGFR: Epidermal growth factor receptor
ER: Endoplasmic reticulum
GLP1: Glypican-1
HB-EGF: Heparin-binding growth factor
HNSCC: Head and neck squamous cell carcinoma
IBD: Inflammatory bowel disease
IGF-1: Insulin growth factor 1
L-1R2: Interleukin 1 receptor 2
IL23R: Interleukin 23 receptor
iPSC: Human-induced pluripotent stem cell
JAG: Jagged 1
LRIG1: Leucine-rich repeats and immunoglobulin-like domains 1
4mut: Prodomain of ADAM17
NRG1: Neregulin-1
NK: Natural killer
PD-1: Programmed cell death ligand
PTP*α*: Protein tyrosine phosphatase alpha
RA: Rheumatoid arthritis
SDC1: Syndecan-1
SDC4: Syndecan-4
TGF*α*: Transforming growth factor alpha
TGI: Tumor growth inhibition
TKI: Tyrosine kinase inhibitor
TNC: Triple-negative breast cancer
TNF*α*: Tumor necrosis factor alpha
VEGFR: Vascular endothelial growth factor receptor 1.
